# Editorial: Home-based training to reduce upper limb functional impairment post-stroke

**DOI:** 10.3389/fneur.2023.1309954

**Published:** 2023-10-24

**Authors:** Paulette van Vliet, Meredith Tavener, Frederike M. van Wijck, Margit Alt Murphy

**Affiliations:** ^1^School of Health Sciences, College of Health, Medicine and Wellbeing, The University of Newcastle, Callaghan, NSW, Australia; ^2^School of Medicine and Public Health, College of Health, Medicine and Wellbeing, The University of Newcastle, Callaghan, NSW, Australia; ^3^School of Health and Life Sciences, Glasgow Caledonian University, Glasgow, United Kingdom; ^4^Department of Clinical Neuroscience, Institute of Neuroscience and Physiology, University of Gothenburg, Sahlgrenska Academy, Gothenburg, Sweden

**Keywords:** stroke, upper limb, functional impairment, home, rehabilitation, training

Home is where much of the upper limb recovery after stroke takes place. This can be due to a variety of factors such as limits on the amount of inpatient rehabilitation, precedence of lower limb training in hospital, or the length of time it can take to regain functional arm and hand use. A trend toward moving inpatient rehabilitation services into the community and COVID-19 have also increased reliance on home-based rehabilitation. This raises many questions about home-based rehabilitation for the upper limb including optimal content and delivery, effects, and stroke survivors' experiences.

The effectiveness of home-based self-managed low-cost upper limb programs, when compared to other home programs, was investigated in a systematic review by Westlake et al. This showed little evidence of superiority of a purposively designed upper limb program over a more conventional one. The main reason for this neutral effect could be the low contrast between the groups. The review identified, however, eight promising interventions covering a range of home programs, such as the GRASP program, neuromotor stimulation, video games and use of a Music Glove or robotic arm.

In contrast, Toh et al. compared home programs to conventional programs delivered in clinical settings. Interestingly, the non-technology programs (constraint-induced movement therapy, therapeutic exercises, and goal-oriented task-specific training) were superior or equal to conventional intervention provided either at clinic or home—with the exception of electrical stimulation, which provided significantly more improvement in upper limb function than treatment without electrical stimulation. These reviews show that home therapy when provided in a well-structured way using sufficient contrast can be beneficial in improving upper limb function and activity, but evidence to point out which specific intervention is most effective is limited.

A fundamental question is how best to facilitate engagement in rehabilitation after stroke. This is necessary for stroke survivors to build new routines that facilitate transfer of what has been learned in a rehabilitation program to meaningful activities in their daily lives. The papers in this Research Topic contribute to a deeper insight in the complexities of what facilitating engagement in rehabilitation may involve. They highlight that this needs to be carefully co-created, with input from the stroke survivor and their family/carers (if available) and professionals, and monitored.

A crucial factor for engaging people with their home-based rehabilitation- especially where there is minimal or no professional input—is their intrinsic motivation (Schnabel et al.). This is needed to generate the drive to initiate, maintain and—where possible—progress practice toward an individual's personal goals.

It is important that therapists take time to work with stroke survivors (and family/carers if available and appropriate) to gain an understanding of their views on the life roles and goals they wish (or need) to return to after their stroke, as well as salient physical and social aspects of their home environment. Any rehabilitation activities need to be clearly linked to their needs and daily lives in order to spark the intrinsic motivation necessary to initiate and sustain practice (Schnabel et al.).

A key requirement is to set the challenge, posed by the rehabilitation exercises and/or activities, at the appropriate level (De Jesus Ramos Muñoz et al.). Findings in the papers in this Research Topic underscore the Challenge Point Hypothesis (De Jesus Ramos Muñoz et al.) which posits that skill acquisition is maximized under an optimal challenge level. In cases where there is no professional input, rehabilitation technology needs to be designed in such a way that individual users can easily select (and then progress) the appropriate challenge level that yields a success rate of more than 90%—but <100% (De Jesus Ramos Muñoz et al.). Mastery experience is necessary for stroke survivors to boost their self-efficacy which, in turn, is needed to build new practice routines (Schnabel et al.), and to transfer the learning to other skills they need. Mixed reality gaming interventions, such as the one piloted by Ham et al., are a promising development as they combine an immersive environment, where challenge levels can be carefully controlled and progressed, with activities that involve actual objects.

Persevering is associated with initiating self-practice (i.e., “getting started again”), as shown by the work by De Jesus Ramos Muñoz et al. Steady engagement is better than practicing too much too early, which may induce fatigue, adverse effects or boredom. Steady engagement requires discipline, which may be more difficult for those who lack regular family—and/or work commitments (Schnabel et al.). Other factors (e.g., anxiety, depression, lacking clarity about one's rehabilitation goals) may also impede engagement in self-managed practice. Particularly in these circumstances, input from professionals may be pivotal (Schnabel et al.).

Regular follow-up, either by a professional or through logs, is necessary to review how well stroke survivors engage in their rehabilitation. Daily logs that are reviewed on a weekly basis were shown to be most commonly used (De Jesus Ramos Muñoz et al.), but further research is needed to work out which formats are least burdensome. Self-report is common, but it is well known that this may be confounded by several factors (including memory), and therefore further research on objective measures of engagement (e.g., through technology is an important line of further research (De Jesus Ramos Muñoz et al.).

It will be exciting to learn the findings from the two studies for which the protocols were described in this Research Topic; the protocol by van Vliet et al. focuses on progressive functional task-specific practice in the home, with therapist visits being tapered down over the course of the intervention to facilitate engagement in self-managed practice. The protocol by Christie et al. comprises functional task practice and shaping techniques that align with the Challenge Point Hypothesis, as well as a transfer package to support engagement by stroke participants.

Taken together, the work comprised within this Research Topic highlights the importance of collaborative working between healthcare professionals, stroke survivors and family/carers, as well as rehabilitation technology designers, to ensure that rehabilitation programs are meaningful, appropriate and engaging, to ultimately enable stroke survivors to achieve their goals.

## Author contributions

PV: Conceptualization, Writing—original draft, Writing—review and editing. MT: Conceptualization, Writing—original draft. FW: Conceptualization, Writing—original draft, Writing—review and editing. MA: Conceptualization, Writing—original draft, Writing—review and editing.

